# High prevalence of Helicobacter pylori infection in Behcet’s disease

**DOI:** 10.1186/1471-230X-14-58

**Published:** 2014-03-31

**Authors:** Kamran B Lankarani, Mohammad Reza Ravanbod, Elham Aflaki, Mohammad Ali Nazarinia, Akbar Rajaee

**Affiliations:** 1 Health policy Research Center, Shiraz University of Medical Sciences, P.O. Box 71345–1414, Shiraz, Islamic Republic of Iran; 2 Bushehr University of Medical Sciences, Bushehr, Islamic Republic of Iran; 3 Rheumatology Research Center, Shiraz University of Medical Sciences, Shiraz, Islamic Republic of Iran

**Keywords:** Helicobacter pylori, Behcet’s disease, Pathogenesis, Urea breath test

## Abstract

**Background:**

Behcet’s disease (BD) is a multisystem disease of unknown etiology. There are several clues which may indicate an ethiopathogenesis role for Helicobacter pylori infection in this disease.

**Methods:**

In a case control study in an out patient department, 48 patients with BD were compared to age, sex matched controls regarding presence of H. pylori infection by serology and urea breath test (UBT).

**Results:**

Ongoing H. pylori infection was more prevalent among patients with BD using result of UBT with odds ratio of 3.1 (95% CI: 1.34 – 7.26, PV < 0.001).

**Conclusion:**

H. pylori infection may have a role in the pathogenesis of BD.

## Background

Behcet’s disease is a systemic disease of unknown etiology affecting multiple organs including skin, eye, mucosa of gastrointestinal and genitourinary tract, joints, endothelial surface of arteries and veins [1].

The disease is most common along the silk route but has been reported from almost all continents [1,2].

It has clear differences with many other rheumatologic disorders which have autoimmune basis in their pathogenesis and the role of environmental factors were considered prominent in this disease [2-4].

There are several clues which may indicate an ethiopathogenesis role for Helicobacter pylori infection in this disease. This infection is most prevalent in areas where BD is common [5-7]. The geographic distribution of the most grave complication of this infection, gastric cancer have many similarities to BD [8,9].

This study was designed to find any possible relation between H. pylori infection either current or in past with BD.

## Methods

Patients with a definite diagnosis of BD, based on international study group criteria [10] who were in regular follow-up for at least one year in Behcet’s disease Clinic in Motahari Clinic, Shiraz University of Medical Sciences, Islamic Republic of Iran, were studied. Clinical manifestations and organ involvement along with demographic features were recorded.

For the control group, age and sex matched healthy asymptomatic people who were referred for routine check-ups as a part of their regular health surveillance were studied. Specific questions regarding presence of any gastrointestinal compliant and/or previous or current history of gastrointestinal diseases were obtained from both control and study groups and any subject with positive history were excluded. Other exclusion criteria in both cases and controls were: pregnancy, use of proton pump inhibitor or antibiotics in the past month, any co morbid condition such as renal failure, liver disease, chronic obstructive pulmonary disease, congestive heart failure, age under 18 or over 65 years.

Anti Helicobacter Pylori antibody IgG were assayed by ELISA (RADIM, Iran) in both groups. Urea breath test (UBT) was also done for all cases and controls as described elsewhere with carbon 14 after overnight fasting and tooth brushing [11]. If the result of any of these tests were positive, patients were considered infected with H. pylori.

The study design was approved by the ethics committee of the Shiraz University of Medical Sciences.

Informed consent from both groups was taken. Odds ratio, with 95% confidence interval Chi Square (Pearson) and X2 Fisher Exact tests were calculated when appropriate.

## Results

48 patients (17 males) with BD fulfilled our criteria and were studied. The same number of healthy people was enrolled in the control group.

Both groups were comparable regarding mean age and sex ratio (Table 1).

**Table 1:** Demographic features and Helicobacter pylori status of patients with BD and control group

	**Mean age ± SD**	**Male**	**Serology positive**	**UBT positive**	Cumulative number of HP infection^*^
**Case**	33.2±8.6	17	29	34	37
**Controls**	32.7±8.8	17	23	20	27
**PV**	NS	NS	NS	< 0.01	< 0.05

*Total number of HP infection when either serology and or UBT were positive.

H. pylori infection as determined by positive UBT was more prevalent in patients with BD, but the results of serology were not significantly different between the two groups.

When a cumulative number of infected patients using two methods was utilized, the odds ratio for H. pylori was 2.4026 (95% CI: 0.99 – 5.828, PV: <0.05).

When the result of UBT alone was considered, the ratio was 3.1 (95% CI: 1.34 – 7.26, PV < 0.001).

The odds ratios of H. pylori infection for each major organ involvement in patients with BD are shown in Table 2, using a cumulative number of infected patients.

**Table 2:** Correlation of clinical findings in BD with HP infection

	**Total number**	**HP infected**	**OR**	**95% CI for OR**	**P value**
**Positive pathergy test**	13	10	0.99	0.22-4.49	0.987
**Urogenital ulcers**	23	18	1.14	0.30-4.39	0.852
**Arthritis**	9	8	2.76	0.31-24.89	0.366
**Uveitis**	12	7	0.28	0.07-1.19	0.084
DVT*	4	3	0.88	0.08-9.44	0.918
**Acneiform rash**	16	14	2.74	0.52-14.55	0.237
**Erythema nodosum**	4	3	0.88	0.08-9.44	0.918

*DVT: Deep Vein Thrombosis.

There was only one patient with neurobehcet in this series who was H. pylori infected, therefore the odds ratio could not be calculated for this manifestation.

## Discussion

Despite description of BD for more than 2000 years, the pathogenesis of this disease is not clear [3,12,13]. Both genetic and environmental factors are considered to have ethiopathogentic role in BD [14]. It has been shown that Turkish immigrants to Germany have higher incidence of BD compared to native Germans but the incidence of BD is much lower than Turkish population living in Turkey [13,15]. The same pattern is reported for Japanese immigrants to Hawaii, mainland United States and south America [13,16].

Among the environmental factors, the role of infectious agents have been proposed [13]. Viral, bacterial, mycobacterial and even fungal agents have been studied in these patients with variable results.

Several investigators have studied the role of H. pylori infection in BD. Almost all of these studies are from Turkey [17-20]. Avci and colleagues from Turkey probably were the first group to publish on this issue [17]. In their serologic case control study, they did not find no higher prevalence of H. pylori infection in BD compared to control group but H. pylori eradication in a small group of their patients resulted in alleviation of symptoms. Ersoy and Cakmak and their colleagues in two other different studies from Turkey could not find any relation between BD and H. pylori in endoscopic biopsies [18,19]. Another group from Turkey reported significantly higher prevalence of cytotoxin-associated gene A positivity in BD [21]. In the latter study prevalence of anti H. pylori immunoglobulin G antibody was not significantly higher in BD which is in concordance to previous studies but patients’ symptoms improved after H. pylori eradication like Avci’s report.

Interestingly there are only case reports on association of H. pylori infection and BD from Japan [22].

In this study from Iran, with a prevalence BD of 80 per 100,000 of inhabitants [7], we found higher prevalence of H. pylori infection in patients with BD. This relation was more prominent when result of UBT was used with OR of 3.1 (95% CI: 1.34 – 7.26, PV < 0.001). Using serology, there was no significant difference between case and control group regarding prevalence of H. pylori infection as was reported by previous researchers.

The reason that serologic evidence of H. pylori infection was not different between two groups might be related to the fact that serology can not differentiate between ongoing infection or previous exposure [23]. It seems BD has a correlation with ongoing infection, as even in previous studies who failed to show a correlation between infection and BD, there were symptomatic improvement with H. pylori eradication in those who were infected [17,21]. Although two previous studies using endoscopic biopsies were not able to show such a relation [18,19], but we should note that there are several intervening factors which may result in false negativity of endoscopic biopsies for H. pylori infection detection. These include inadequate specimens, use of proton pump inhibitors and other acid suppressive agents, recent use of antibiotics among others [23]. In our study we strictly excluded patients who had factors which could interfere with result of UBT and this may explain the different results.

Although the odds ratio for H. pylori infection using cumulative number of infected patients by both UBT and serology was 2.4026 with a p value of less than 0.05. This may be related to relatively small size of our study group [24].

Whether any specific presentation of BD is related to H. pylori infection, has been investigated in few studies. Researchers from Turkey did not find any relation between deep vein thrombosis and H. pylori infection in BD [20]. In our own study we could not find statistically significant correlation between H. pylori infection and BD manifestations, but it seems the limited number of our cases have been contributing. Larger studies are needed to investigate such relation.

The possible mechanism of action of bacterial infections including H. pylori in the pathogensis of BD may involve molecular homology of bacterial antigens and human heat shock protein (HSP) which is now proposed as the possible antigen in BD [3,4]. This antigen is expressed outside of cellular membrane in response to shock and other stresses and physiologic stimuli. Elevated HSP and antibody against this protein have been shown in sera of patients with BD [25]. The mucosal expression of HSP was also found to be higher in BD [26]. There is homology between bacterial antigens and human HSP. Although much of this homology has been reported for Streptococcus sanguis [27], the same homology has also been reported for H. pylori [28]. This would stimulate δγ T cells. This stimulation would be further augmented with exposure to human HSP and start a cascade of proinflammatory reactions.

## Conclusions

In conclusion our study from Iran using UBT could reveal a correlation between ongoing H. pylori infection and BD in contrary to all previous reports which had some limitations in their methodologies. To find a correlation between H. pylori infection and specific manifestations of BD, larger studies are needed.

## Competing interests

The authors declare that they have no competing interests.

## Authors’ contributions

KBL developed the idea, arranged the logistic for the study, contributed in data analysis and preparation of manuscript. MRR, MAN, EA and contributed in data gathering and analysis of data and preparation of the manuscript. AR contributed to data gathering and preparation of the manuscript. All authors read and approved the final manuscript.

## Pre-publication history

The pre-publication history for this paper can be accessed here:

http://www.biomedcentral.com/1471-230X/14/58/prepub
